# Altmetric Attention Scores and Citations of Published Research With or Without Preprints

**DOI:** 10.1001/jamanetworkopen.2024.24732

**Published:** 2024-07-26

**Authors:** Seth Zissette, Anant Gautam, Harlan M. Krumholz, Joseph S. Ross, Joshua D. Wallach

**Affiliations:** 1 Department of Epidemiology, Rollins School of Public Health, Emory University, Atlanta, Georgia; 2 South Forsyth High School, Cumming, Georgia; 3 Section of Cardiovascular Medicine, Department of Internal Medicine, Yale School of Medicine, New Haven, Connecticut; 4 Section of General Internal Medicine, Department of Internal Medicine, Yale School of Medicine, New Haven, Connecticut

## Abstract

This cross-sectional study assesses how frequently research articles published in the clinical journals with high impact factors are preprinted and whether preprinting is associated with changes in media attention and citation counts.

## Introduction

Use of preprints, defined as preliminary research reports that have not undergone peer review, in clinical and health science research has increased in recent years, partly because of the COVID-19 pandemic.^1,2^ Although high-impact clinical journals have publication policies supportive of preprints,^3^ concerns remain that posting a preprint before submission to a peer-reviewed journal may jeopardize publication, especially if the preprint generates media attention and citations.^4^ While biology articles with corresponding preprints have received greater attention than those without,^5^ little is known about the highest-impact clinical research. Therefore, we aimed to assess how frequently research articles published in the highest-impact clinical journals are preprinted and whether media attention and citations differed between articles with and without corresponding preprints.

## Methods

In accordance with the Common Rule, this cross-sectional study was exempt from ethics review and informed consent because it used public nonidentifiable data. We followed the STROBE reporting guideline.

We identified 25 high-impact journals according to InCites Journal Citation Reports, including the 6 general and internal medicine journals and the 2 clinical medicine journals across 9 subspecialities with the highest impact factors (Table 1). We also included *JAMA Network Open* as it publishes general and subspecialty clinical articles. We searched PubMed for all records of these journals indexed in 2022 (after the COVID-19 pandemic had largely subsided) and manually identified original research articles. First, we identified preprints automatically linked to published articles in the sample using the bioRxiv/medRxiv application programming interface (API). To locate preprints missed by the API or posted on other platforms, we used WebScrapingAPI to conduct Google searches of published article titles and screened the first 5 records mentioning medRxiv, bioRxiv, or Social Science Research Network. Second, we identified the Altmetric Attention Score (Altmetric API) and citations (Dimensions API) for each article as of March 2024.

**Table 1.** Preprints of Clinical Research Subsequently Published in 25 High-Impact Journals

Journal; 2022 Impact factor^a^	Original research articles			Original research articles with a corresponding preprint		
	No.	Altmetric Attention Score, median (IQR)	Citations, median (IQR)	No. (%)	Altmetric Attention Score, median (IQR)	Citations, median (IQR)
**General and internal medicine journals**						
*The New England Journal of Medicine;* 158.5	273	642.0 (367.9-1062.8)	76.0 (36.0-174.0)	41 (15.1)	1370.0 (478.5-3947.1)	237.0 (49.0-341.0)
*The Lancet;* 168.9	197	235.6 (73.6-767.0)	47.0 (15.0-119.0)	15 (7.6)	233.2 (95.1-889.8)	127.0 (20.5-292.0)
*JAMA;* 120.7	254	218.4 (79.9-482.2)	21.0 (8.3-46.3)	8 (3.1)	486.7 (330.7-1244.3)	50.5 (18.3-129.3)
*The BMJ;* 107.7	150	238.0 (73.2-641.3)	21.5 (11.0-48.0)	26 (17.3)	333.7 (96.2-875.5)	53.0 (22.5-67.3)
*Annals of Internal Medicine;* 39.2	160	278.4 (81.6-400.3)	12.0 (4.8-23.0)	12 (7.5)	252.8 (306.6-370.5)	42.0 (22.0-60.0)
*JAMA Internal Medicine;* 39.0	140	136.8 (65.5-381.3)	13.5 (6.0-35.3)	5 (3.6)	873.7 (264.1-1638.6)	75.0 (47.0-78.0)
*JAMA Network Open;* 13.8	1825	38.8 (15.3-159.7)	7.0 (3.0-13.0)	95 (5.2)	71.7 (25.5-321.3)	11.0 (6.0-24.0)
**Clinical medicine subspecialty journals**						
Cardiac and cardiovascular systems						
*European Heart Journal;* 39.3	146	74.6 (36.9-118.7)	21.5 (13.0-33.0)	2 (1.4)	1200.4 (638.6-1762.1)	28.5 (24.8-32.3)
*Circulation;* 37.8	219	43.8 (18.7-117.0)	16.0 (9.0-33.0)	14 (6.4)	39.3 (27.3-80.1)	18.0 (13.0-23.8)
Endocrinology and metabolism						
*The Lancet Diabetes & Endocrinology;* 44.5	47	96.2 (53.1-186.8)	28.0 (17.0-53.0)	0	NA	NA
*Diabetes Care;* 16.2	330	11.1 (3.6-26.2)	7.0 (4.0-13.0)	6 (1.8)	75.4 (34.6-176.1)	15.0 (10.3-19.0)
Gastroenterology and hepatology						
*The Lancet Gastroenterology & Hepatology;* 35.7	64	36.4 (21.3-83.9)	28.0 (14.8-49.0)	4 (6.3)	28.9 (8.9-96.9)	34.0 (25.8-137.5)
*Gastroenterology;* 29.4	171	22.2 (7.3-43.5)	14.0 (6.0-27.5)	12 (7.0)	41.4 (31.0-51.7)	18.0 (12.8-33.8)
Hematology						
*The Lancet Haematology;* 24.7	54	31.1 (17.0-48.2)	17.0 (10.3-24.8)	3 (5.6)	62.1 (43.3-83.5)	15.0 (12.0-23.5)
*Blood;* 20.3	301	8.5 (2.5-16.1)	14.0 (8.0-28.0)	24 (8.0)	9.5 (2.9-19.6)	11.5 (5.0-25.0)
Infectious disease						
*The Lancet Infectious Diseases;* 56.3	157	110.1 (33.0-474.1)	28.0 (12.0-64.0)	41 (26.1)	315.8 (103.8-706.8)	48.0 (23.0-100.0)
*Journal of Infection;* 28.2	335	3.9 (1.0-14.3)	5.0 (2.0-9.0)	32 (9.6)	4.1 (2.5-12.2)	6.0 (2.0-14.0)
Urology and nephrology						
*Kidney International;* 19.6	145	10.1 (4.3-21.0)	9.0 (6.0-14.0)	14 (9.7)	11.9 (6.2-20.8)	12.5 (8.8-24.3)
*Journal of the American Society of Nephrology;* 13.6	108	20.5 (8.9-45.1)	10.0 (5.0-19.0)	14 (13.0)	29.0 (20.5-52.6)	8.5 (5.3-15.0)
Oncology						
*CA: A Cancer Journal for Clinicians;* 254.7	6	22.0 (19.2-319.7)	11.0 (7.5-19.0)	0	NA	NA
*The Lancet Oncology;* 51.1	118	68.8 (37.5-116.7)	29.5 (16.3-67.3)	1 (0.8)	166.6 (166.6-166.6)	117.0 (117.0-117.0)
Respiratory system						
*The Lancet Respiratory Medicine;* 76.2	80	80.2 (29.6-233.0)	39.5 (19.0-57.5)	11 (13.8)	412.7 (89.8-1721.5)	53.0 (32.0-93.5)
*European Respiratory Journal;* 24.9	220	10.3 (3.5-27.1)	10.0 (4.8-18.0)	20 (9.1)	21.9 (6.6-49.3)	14.5 (10.8-30.8)
Rheumatology						
*Annals of the Rheumatic Diseases;* 27.4	195	19.5 (6.1-40.2)	11.0 (6.5-26.0)	15 (7.7)	11.7 (29.5-47.9)	23.0 (8.0-42.0)
*The Lancet Rheumatology;* 25.4	44	43.3 (19.2-89.5)	15.5 (8.0-29.0)	10 (22.7)	79.1 (44.1-132.1)	14.0 (7.5-25.0)
Total	5739	40.2 (11.6-265.0)	11.0 (5.0-26.0)	425 (7.4)	85.0 (20.6-456.9)	21.0 (9.0-53.0)

Abbreviation: NA, not applicable.

^a^ Includes 6 general and internal medicine journals (and *JAMA Network Open*) and 2 clinical medicine journals for 9 subspecialties with the highest impact factors according to InCites Journal Citation Reports.

Within each journal with at least 1 article with a preprint, we calculated the differences in median Altmetric scores and citations between articles with and without preprints. We used Wilcoxon signed-rank test to evaluate whether the median of the distribution of the differences between medians across the 25 journals differed from 0. Two-tailed *P* < .05 was considered statistically significant. Data analysis was performed with R, version 4.2.1 (R Project for Statistical Computing).

## Results

Among the 5739 research articles published in 25 journals with high impact factors in 2022, 425 (7.4%) articles in 23 journals had corresponding preprints, ranging from 0 to 41 (26.1%) articles (Table 1). COVID-19–related articles were more likely than non-COVID-19–related articles to have corresponding preprints (257 of 1270 [20.2%] vs 168 of 4469 [3.8%]; *P* < .001).

The median (IQR) difference in medians between articles with and without preprints across journals was not significantly different from 0 for Altmetric Attention Scores (34.1 [6.0-191.0]; *P* = .33) or citations (8.0 [3.0-30.8]; *P* = .31) (Table 2). These findings were consistent even after accounting for time from publication to analysis and when stratified by COVID-19–related and non-COVID-19–related articles.

**Table 2.** Journal-Level Differences in Median Altmetric Scores and Citation Counts Between Articles With and Without Corresponding Preprints

	Articles with corresponding preprint, median (IQR)	Articles without corresponding preprint, median (IQR)	Difference by journal, median (IQR)	*P* value
Overall^a^				
Altmetric Attention Scores	85.0 (20.6 to 456.9)	38.0 (11.1 to 180.2)	34.1 (6.0 to 191.0)	.33
Citations	21.0 (9.0 to 62.6)	11.0 (5.0 to 25.0)	8.0 (3.0 to 30.8)	.31
COVID-19–related articles^b^				
Altmetric Attention Scores	298.6 (59.9 to 857.2)	83.2 (16.0 to 431.4)	45.2 (6.7 to 140.6)	.48
Citations	41.0 (15.0 to 95.0)	14.0 (5.0 to 36.0)	22.8 (12.0 to 34.9)	.29
Non-COVID-19–related articles^c^				
Altmetric Attention Scores	25.3 (8.7 to 63.1)	33.0 (10.8 to 128.6)	4.0 (−6.4 to 29.9)	.78
Citations	12.0 (6.8 to 18.0)	10.0 (5.0 to 22.0)	−1.3 (−3.8 to 3.4)	.82

^a^ *The Lancet Diabetes & Endocrinology* and *CA: A Cancer Journal for Clinicians* were excluded from these analyses because these journals had no articles with corresponding preprints.

^b^ *The Lancet Diabetes & Endocrinology*, *Diabetes Care*, *The Lancet Haematology*, *CA: A Cancer Journal for Clinicians*, and *The Lancet Oncology* were excluded from these analyses because these journals did not publish any COVID-19–related articles with corresponding preprints.

^c^ *Annals of Internal Medicine*, *The Lancet Diabetes & Endocrinology*, and *CA: A Cancer Journal for Clinicians* were excluded from these analyses because these journals did not publish any non-COVID-19–related articles with corresponding preprints.

## Discussion

The finding that 7.4% of published articles in high-impact journals in 2022 had corresponding preprints is similar to estimates in other fields.^5^ However, unlike previous evaluations of publications and corresponding bioRxiv preprints,^5,6^ this study found no differences in Altmetric Attention Scores or citations between articles with and without preprints in the first years after publication.

Study limitations include examining only publications in journals with high impact factors and postings in prominent preprint servers. Furthermore, it is possible that authors are more likely to preprint their most important articles and that the published version of these articles would have received more media attention and citations if they had not been preprinted. However, although not all preprints will subsequently be published in high-impact journals, the results suggest that, among published articles with corresponding preprints, preprinting is not associated with less media attention and lower citation counts.

## Notes

Supplement.
Data Sharing Statement
