# Supplementary material for: Altmetric Attention Scores and Citations of Published Research With or Without Preprints
Source: JAMA Netw Open. 2024 Jul 26;7(7):e2424732. doi: 10.1001/jamanetworkopen.2024.24732 (PMC11282438; doi:10.1001/jamanetworkopen.2024.24732)
Supplement: Supplement. — Data Sharing Statement [file jamanetwopen-e2424732-s001.pdf]

Data Sharing Statement

Zissette. Altmetric Attention Scores and Citations of Published Research With or Without Preprints. *JAMA Netw Open*. Published July 26, 2024.
doi:10.1001/jamanetworkopen.2024.24732

Data
Data available: Yes
Data types: Data (not involving human participants)
How to access data: The data will be posted on OSF.io upon publication: https://osf.io/y2zvq/
When available: With publication

Supporting Documents
Document types: Statistical/analytic code
How to access documents: The analytic code will be posted on OSF.io upon publication: https://osf.io/y2zvq/
When available: With publication

Additional Information
Who can access the data: Anyone requesting the data
Types of analyses: For any prupose
Mechanisms of data availability: Without investigator support
Any additional restrictions: None
